# HTLV-2 infection in Manaus, Brazil: first description of HTLV-2c subtype in an urban area of the Western Amazon region

**DOI:** 10.1590/0037-8682-0066-2020

**Published:** 2020-11-13

**Authors:** Gemilson Soares Pontes, Hygor Halyson Figueiredo Ribeiro, Diana Mota Toro, José Pereira de Moura, Victor Souza, Maria Edilene Martins de Almeida, Valdinete Alves do Nascimento, Cristovão Alves da Costa, Felipe Gomes Naveca, Mike Santos, Antonio Carlos Rosário Vallinoto

**Affiliations:** 1 Instituto Nacional de Pesquisa da Amazônia, Manaus, AM, Brasil.; 2 Universidade do Estado do Amazonas, Programa de Pós-graduação Stricto Sensu em Hematologia, Manaus, AM, Brasil.; 3 Universidade Federal do Amazonas, Manaus, AM, Brasil.; 4 Fundação Oswaldo Cruz, Instituto Leônidas e Maria Deane, Manaus, AM, Brasil.; 5 Universidade Federal do Pará, Programa de Pós-graduação Stricto Sensu em Biologia de Agentes infecciosos e Parasitários, Belém, PA, Brasil.

**Keywords:** Prevalence, Blood transfusion, Phylogeny, HTLV-2c, Amazon region

## Abstract

**INTRODUCTION:**

We investigated the prevalence of human T-cell lymphotropic virus types 1 and 2 (HTLV-1/2) infection in patients with hematological diseases from the western Amazon region of Brazil.

**METHODS:**

Samples from 306 patients were submitted for the molecular diagnosis of HTLV-1/2 infection by real time PCR (qPCR), with amplification, sequencing, and phylogenetic analysis of the long terminal repeat (LTR) region.

**RESULTS:**

A 29-year-old male carrier of sickle cell anemia with a history of multiple blood transfusions was diagnosed with the HTLV-2c subtype**.**

**CONCLUSIONS:**

This study describes the first known occurrence of HTLV-2c in the urban area of Brazil’s western Amazon region.

Human T-lymphotropic virus 2 (HTLV-2) was described in 1982, after being isolated from a patient with hairy T cell leukemia^1^. Currently, it is estimated that there are almost one million people infected with HTLV-2 worldwide^2^. This retrovirus is predominantly endemic among the Central African pygmy communities and in indigenous populations from the North, Central, and South America^3^ ^-^ ^5^. Phylogenetic analysis of the nucleotide sequences from the transcriptional regulatory region 5’LTR revealed the existence of the following four HTLV-2 subtypes: HTLV-2a, HTLV-2b, HTLV-2c and HTLV-2d^5^ ^-^ ^7^. 

The first evidence of HTLV-2 infection among Amazonian indigenous groups was found in the Kayapo tribe^8^. Later molecular analysis of these isolates provided the first identification of the HTLV-2c^5^ virus subtype. Subsequent studies have confirmed the endemicity of the subtype in the indigenous ethnic groups across the Brazilian Amazon region^9^. HTLV-2c has also been found among urban populations of the eastern Brazilian Amazon region^9^. However, there have been no reports of HTLV-2c reaching urban areas of the western Amazon.

From April 2015 to December 2016, we recruited a randomly selected group of patients from attendees at the outpatient clinic of the Hematology and Hemotherapy Foundation of the Amazon (HEMOAM). All patients were receiving consultation for hematological diseases and only those with a confirmed diagnosis were eligible to participate (Table 1). Peripheral blood samples were collected from 306 patients who signed an informed consent form approved by the HEMOAM ethical committee (approval number: 944.784). Patients were of different ethnicities and socioeconomic backgrounds, with ages ranging from 1 to 92 years old.

**TABLE 1:** Frequency of hematological diseases observed among the study population.

Hematological Diseases	N (%)
Leukemia	134 (43.7)
Anemia (Aplastic, Sickle cell, Hemolytic and other types of anemia)	90 (29.4)
Platelets diseases	39 (12.7)
Lymphoma	22 (7.1)
Hemophilia	9 (2.9)
Hemoglobinopathies	2 (0.65)
Thalassemia	3 (0.98)
Multiple myeloma	3 (0,98)
Myelodysplastic syndromes	2 (0.65)
Polycythemia	1 (0.32)
Spherocytosis	1 (0.32)
**Total**	**306 (100)**

HTLV-1/2 infections were diagnosed from proviral DNA extracted from patients’ peripheral blood mononuclear cells (PBMC). Samples were submitted to the amplification of tax gene sequences conserved across HTLV-1/2 isolates, as has been previously described^10^. Briefly, 200 ng of DNA together with primers (HTV-F5, 5′-CGG ATA CCC IGT CTA CGT GTT T-3′; HTV-R4, 5′CTG AGC IGA IAA CGC GTC CA-3′) and HTLV probe (P-HTV 5′-Reporter dye-FAM-ATC ACC TGG GAC CCC ATC GAT GGA-3′-TAMRA-Quencher dye) underwent qPCR with 2 min at 95ºC, followed by 50 cycles of 15 sec at 95ºC and 1 min at 60°C. The assays were performed in duplicate.

HTLV-2 subtyping was performed through amplification of the 788pb DNA fragment corresponding to the 5’LTR region, as has been described previously^9^. In short, we carried out a nested PCR using 500 ng of DNA and two sets of primers. The first reaction used F-IILTR (5’LTR-2 5’-TCGCGATGACAATGGCGACTAGCCTC-3’) and Long-Gag (5’LTR-2 5’-GGGGGCTTTGGGTATTGGAGTTGGG-3’); Mo 16 (5’LTR-2 5’-GCCTCCCAAGCCAGCCAC-3’) and MSW-Gag (5’LTR-2 5’-GGGAAAGCCCGTGGATTTGCCCCAT-3’) were used in the second reaction. Both reactions involved initial denaturation for 5 min at 94ºC, followed by 35 cycles of 40 sec at 94ºC, 30 sec at 57ºC, and 1 min at 72ºC. 

The DNA was sequenced after cloning the PCR product into a pGEM®-T vector system (Promega Corporation, Madison, WI, USA). The 5’LTR region was sequenced using M13 primers and a BigDye Terminator v3.1 cycle sequencing kit (Thermo Fisher, Waltham, MA, USA). 

Phylogeny inference was performed with MEGA-6 software (Mega Software, Tempe, AZ, USA) applying the neighbor-joining method under dataset encompassing sequences retrieved from GenBank. Robustness of tree topology was evaluated with 1,000 bootstrap pseudoreplicates. 

We diagnosed one HTLV-2 infected patient (0.32%). A similar prevalence has been found in the general population of Brazilian cities such as Belo Horizonte (0.32%) and Rio de Janeiro (0.33%)^11^. Despite these low numbers, reports have indicated that the prevalence of HTLV-1/2 infection tends to be higher in patients with hematological diseases, possibly from blood transfusions^12^ ^,^ ^13^. 

The infected patient (JLM151) is a 29-year-old married male patient from a low-income family, with a high school degree and is a carrier of sickle cell disease. He has lived in Manaus his entire life, declared that he has no tattoo or body piercing, and had never used illicit injectable drugs. The patient has been receiving blood transfusions since early childhood. His spouse and parents refused to be tested for HTLV-1/2 infection. The patient does not have a history of sexually transmitted diseases and did not know about his infection. He was among the patient group with the highest blood transfusion rates in the past 12 months (Figure 1).

**FIGURE 1:**
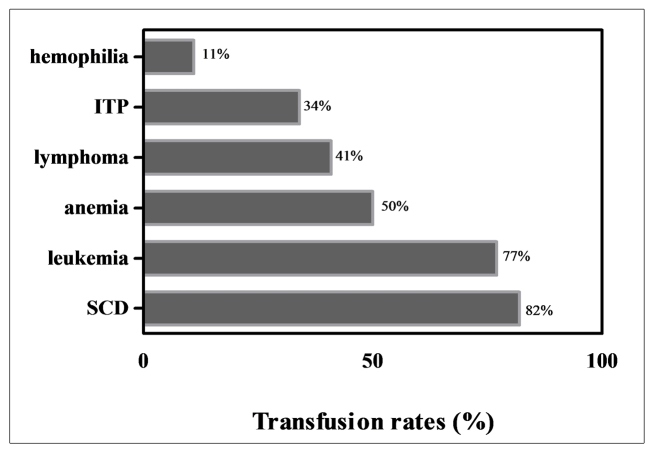
Frequency of blood transfusions of study subjects according to their hematological disease. **ITP:** idiopathic thrombocytopenic purpura; **SCD:** sickle cell disease.

The 611bp 5’LTR fragment sequenced in this study shares over 99% genetic similarity with the HTLV-2c 5’LTR region sequences available in the GenBank database. The JLM151 sample (GenBank accession number MN 266910) was clustered within the subtype HTLV-2c, the same clade of the samples isolated from the Kayapo tribe (AF306731 and AF306731) (Figure 2).The distribution of HTLV-2 subtypes suggests that the viruses were gradually and distinctly dispersed around the world through different migratory events starting from the Africa continent. While subtypes 2a and 2b are especially endemic among the indigenous communities of the North, Central, and South America, HTLV-2c is widely disseminated within native groups from the Brazilian Amazon region^4^ ^,^ ^14^. The first report of HTLV-2c infection in an urban area, the city of Belém, Pará, Brazil, was described in 2003^9^. Since then the subtype has been found in other metropolitan areas in Brazil, but not been previously diagnosed in an urban area of the western Amazon^15^. This study shows the first known circulation of HTLV-2c in Manaus, the largest city of the western Amazon.

**FIGURE 2:**
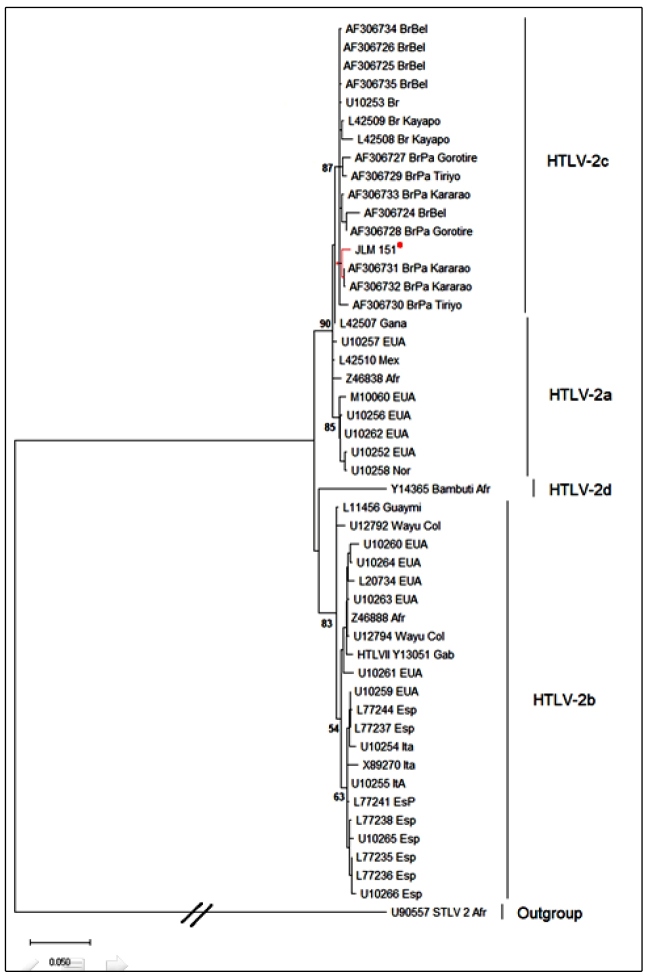
Phylogenetic tree showing the genetic similarity between the 5’ LTR region aligned sequence (611pb) of the HTLV-2 strain isolated in this study, and the sequences of 48 strains from the Genbank database. The tree was constructed using the neighbor-joining method and a bootstrap of 1,000 replicates. The branch of the isolated sample (JLM151) is labeled by a red dot.

Considering that the virus was detected in a patient suffering from sickle cell disease and a frequent recipient of blood transfusions, the infection could plausibly have been transmitted through a transfusion. Brazil has performed serological screening for HTLV-1/2 infection in blood supplies since 1993. However, a study conducted at blood centers from 2007-2009 estimated a residual transfusion risk of 5.0 per 10^6^ per blood product^13^. We could not determine whether the HTLV-2c infection was transmitted by blood transfusion as we were not able to trace all blood products the infected patient had received over the years. Furthermore, neither the patient’s wife nor his parents agreed to be tested for HTLV-1/2 infection, which would have allowed us to evaluate the possibility of sexual or vertical transmission. 

In conclusion, this study demonstrated the prevalence of the HTLV-2c virus in a new geographic area of the western Amazon region. The emergence of HTLV-2c in new urban areas points out the need for expanded public health surveillance of HTLV-1/2 infections. Better and updated information regarding the epidemiology of HTLV-1/2, especially related to the risk of transmission through transfusion, must be regularly monitored by blood centers to guarantee the safety of vital blood transfusions. 
